# Metabolic Reprogramming in Mitochondria of Myeloid Cells

**DOI:** 10.3390/cells9010005

**Published:** 2019-12-18

**Authors:** Hao Zuo, Yihong Wan

**Affiliations:** 1 Department of Pharmacology, The University of Texas Southwestern Medical Center, Dallas, TX 75390, USA; 2 Harold C. Simmons Comprehensive Cancer Center, The University of Texas Southwestern Medical Center, Dallas, TX 75390, USA; 3 Hamon Center for Regenerative Science and Medicine, The University of Texas Southwestern Medical Center, Dallas, TX 75390, USA

**Keywords:** metabolism, mitochondria, myeloid cells, macrophages, glycolysis, oxidative phosphorylation, Krebs cycle, electron transport chain, inflammation

## Abstract

The myeloid lineage consists of multiple immune cell types, such as macrophages, monocytes, and dendritic cells. It actively participates in both innate and adaptive immunity. In response to pro- or anti-inflammatory signals, these cells undergo distinct programmed metabolic changes especially in mitochondria. Pro-inflammatory signals induce not only a simple shift from oxidative phosphorylation to glycolysis, but also complicated metabolic alterations during the early and tolerant stages in myeloid cells. In mitochondria, a broken Krebs cycle leads to the accumulation of two metabolites, citrate and succinate, both of which trigger pro-inflammatory responses of myeloid cells. A deficient electron transport chain induces pro-inflammatory responses in the resting myeloid cells while it suppresses these responses in the polarized cells during inflammation. The metabolic reprogramming in mitochondria is also associated with altered mitochondrial morphology. On the other hand, intact oxidative phosphorylation is required for the anti-inflammatory functions of myeloid cells. Fatty acid synthesis is essential for the pro-inflammatory effect and glutamine metabolism in mitochondria exhibits the anti-inflammatory effect. A few aspects of metabolic reprogramming remain uncertain, for example, glycolysis and fatty acid oxidation in anti-inflammation. Overall, metabolic reprogramming is an important element of immune responses in myeloid cells.

## 1. Introduction

Metabolic reprogramming is an essential response for cells to various environmental and cellular stresses. Immune cells have common metabolism pathways as other cell types and they also have their unique metabolic changes in innate and adaptive immune responses. The history of studying the metabolism in immune cells (termed as immunometabolism) is longer than half a century, yet not until just a decade ago did researchers start to zoom in to the metabolic reprogramming during the differentiation and activation of immune cells [1,2,3]. Now, accumulating evidence demonstrates metabolic reprogramming as a key driver of immune responses of all immune cell types including macrophages, dendritic cells (DCs), and T cells [3,4].

The myeloid lineage is differentiated from the common myeloid progenitor, which is derived from hematopoietic stem cells in the bone marrow. The myeloid lineage includes macrophages, monocytes, neutrophils, eosinophils, basophils, erythrocytes, megakaryocytes, mast cells, and a subclass of DCs [5]. All these cells are widely distributed in all tissues through circulation and actively participate in various biological activities such as inflammation and wound repair. The myeloid cell lineage plays critical roles in both innate and adaptive immunity, and thus, together with the lymphoid lineage (natural killer cells, B cells, and T cells), constitutes a complete immune network in mammals.

Mitochondria are the central energy producers in cells due to their ability of generating numerous ATP from metabolizing fatty acids and the glycolytic product pyruvate through the Krebs cycle (also called TCA cycle) and the oxidative phosphorylation (OXPHOS). Another important role of mitochondria in the myeloid cells is producing reactive oxygen species (ROS) and metabolites to induce pro-inflammatory response and clear infection [3,6]. Mitochondria are also dynamic organelles constantly undergoing fusion and fission to maintain normal functions of mitochondria and regulate metabolism [7].

Some myeloid cells are divided into pro-inflammatory subtypes and anti-inflammatory subtypes, such as M1 and M2 macrophages, and N1 and N2 neutrophils [8]. In vitro, M1 macrophages are differentiated in the presence of IFNγ and Toll-like receptor (TLR) ligands, such as lipopolysaccharide (LPS), while M2 macrophages are induced by IL-4. These two subtypes have distinct metabolism remodeling phenotypes [3]. M1 macrophage polarization is believed to induce a metabolic shift from OXPHOS to aerobic glycolysis (also called Warburg effect), while M2 macrophage polarization relies on OXPHOS [3]. However, recent studies provide new concepts and more details for these phenotypes. In this review, we summarize recent findings and reveal a complex network of metabolic reprogramming in the mitochondria of myeloid cells.

## 2. Glycolysis and Oxidative Phosphorylation

The common opinion of the metabolism reprograming during the activation of myeloid cells by pro-inflammatory signals, such as LPS, includes a glycolytic burst and a suppressed OXPHOS [3]. To achieve these phenotypes, LPS activates two metabolic regulators, the mammalian target of rapamycin (mTOR) and Hypoxia-inducible factor-1α (HIF1α) [9], and suppresses AMP-activated protein kinase (AMPK) [10]. However, recent findings have demonstrated more complicated changes of these two events after LPS-induced activation (Table 1). Based on recent findings, the effect of LPS on glycolysis and OXPHOS can be divided into three periods: early response, sustained response, and tolerant response.

### 2.1. Early Response

As shown in Table 1, glycolysis is enhanced in the early response period (0–4 h) in macrophages, monocytes and DCs, but the changes of OXPHOS are not consistent among different studies. Real-time changes of glycolysis can be measured by lactate release and extracellular acidification rate (ECAR), and OXPHOS is measured by oxygen consumption rate (OCR).

During the early response, one report demonstrates that LPS reduces real-time OCR in macrophages [11]. However, some other real-time measurements of OCR in murine bone marrow-derived macrophages (BMDMs), human monocyte-derived macrophages (hMDMs) and DCs show that LPS causes neither increase nor reduction of OXPHOS during the first 2 h [15,16,21]. Moreover, LPS is also reported to cause a transient increase of OXPHOS within 1 or 6 h [19,20]. In addition, measurements of OCR at 0.5, 1, or 4 h after LPS treatment indicate elevated OXPHOS at these time points [12,13,14,18]. Whether LPS stimulation leads to a transient increase and/or delayed decrease of OXPHOS in the myeloid cells needs more evidence.

The molecular mechanism of the glycolysis burst in the early response involves TBK1-IKKε and Akt-dependent activation of the glycolytic enzyme hexokinase [21]. Mechanisms of the possible transient increase of OXPHOS by LPS are investigated as well. LPS-induced OXPHOS depends on glucose oxidation, which also supports the availability of acetyl-CoA for histone acetylation at the promoters of pro-inflammatory cytokines within 2 h [13], although acetyl-CoA is also reported to be reduced by LPS [11]. By global metabolite profiling, Glycerol 3-phosphate shuttle (GPS) pathway is identified as the top-upregulated pathway by LPS [13]. This pathway can shuttle electrons from cytosolic glycolytic NADH to FADH_2_ for fueling the mitochondrial electron transport chain (ETC). LPS increases the activity of the GPS enzyme mitochondrial glycerol 3-phosphate dehydrogenase (GDP2) to drive mitochondrial respiration [13]. GDP2 knockout (KO) blocks the increase of OCR by LPS in BMDMs [13]. In addition, mitochondrial Complex III (CIII) inhibitor suppresses ROS production in the first hour of IFNγ/LPS treatment [14]. Revisable transient inhibition of CIII for the first one hour is sufficient to block the pro-inflammatory cytokine production during the entire 18-h treatment of IFNγ/LPS [14]. Therefore, normal CIII activity in the early response is required for myeloid cell activation.

### 2.2. Sustained Response

LPS treatment (1–100 ng/mL) for 4–24 h results in increased glycolysis and decreased OXPHOS (Table 1). Transcriptome analysis shows that LPS treatment up-regulates the expression of glycolytic genes and down-regulates the expression of OXPHOS genes [12,17,18,23]. The metabolite profile of LPS-induced myeloid cells reveals accumulated glycolytic intermediates and intermediates of the Krebs cycle, including citrate and succinate [12,15,18]. LPS induced glycolysis is mediated by the PI3K/Akt pathway and HIF1α stabilization, and antagonized by AMPK [12,32]. LPS treatment also triggers the nuclear translocation of the glycolytic enzyme PKM2 to form a complex with HIF1α and regulate glycolysis and pro-inflammatory cytokine production [22]. LPS increases mTOR-mediated inducible nitric oxide synthase (iNOS), and subsequent nitric oxide (NO) production inhibits OXPHOS [20,33].

The metabolic alterations in different conditions (dose, ligand and cell type) may be very complicated. Treatment of 0.1 ng/mL LPS for 24 h in monocytes induces an elevation of both glycolysis and OXPHOS [18]. Treatment of 10 ng/mL LPS for 12 h also induces a slight increase of OXPHOS without modulating glycolysis [25]. LPS is the antigen from Gram-negative bacteria (e.g., *E. coli*) and activates TLR4 receptors of immune cells. Treatment of Pam_3_CysSK_4_ (P3C, TLR2 ligand), poly(I:C) (TLR3 ligand) or lysates from *E. coli*, *S. aureus* and *M. tuberculosis* lead to both increased glycolysis (measured by ECAR and lactate production) and increased or unchanged OXPHOS (measured by OCR, SRC and the activity of several enzymes in the ETC) [18]. However, in another report, treatment of P3C, poly(I:C), R848 (TLR7/8 ligand), and CpG (TLR9 ligand) decreased OCR in DCs [20]. The metabolic changes in human and murine macrophages may be quite different, because LPS-stimulated mouse BMDMs had increased glycolysis and decreased OXPHOS as most other reports, but LPS stimulated hMDMs had a slightly decreased glycolysis and unaltered OXPHOS [27]. Microglia functions as resident macrophages in the central nerves system. Similar Warburg effect was observed in microglia after 100 ng/mL LPS treatment for 24 h, but 100 ng/mL LPS treatment for 6 h or 50 ng/mL LPS treatment for 6 or 24 h increased both glycolysis and OXPHOS [35]. Although the metabolic reprogramming in the myeloid cells has been studied extensively, further investigations on the antigen diversity, the dosage dependent effect and immune cell type dependent responses are necessary.

### 2.3. Tolerant Response

A low dose of LPS pretreatment induces a tolerant state of monocytes and macrophages which are unable to respond to further LPS challenge. The tolerance leads to suppressed immune response upon the subsequent stimulation by the lethal dose of LPS, thus prevents cytokine storm, tissue damage and mortality from septic shock [37]. In vitro, tolerant monocytes and macrophages have reduced gene expression related to glycolysis, OXPHOS, and cytokine production after a second LPS stimulation in comparison to stimulated non-tolerant cells [13,29,36]. The altered metabolism in tolerant myeloid cells is due to an impaired mTOR pathway [29] and GDP2-mediated reverse electron transport (RET), which is the consequence of overwhelming the electron transport chain (ETC) [13]. There is a negative feedback regulation that LPS induces the expression of anti-inflammatory IL-10 and IL-10 in turn inhibits glycolysis and preserves OXPHOS by down-regulating NO production, mTOR activity, glycolytic enzyme expression and preventing the accumulation of dysfunctional mitochondria [15,26].

### 2.4. Glycolysis and OXPHOS in M2 Macrophages

IL-4 has very little effect on glycolysis and no effect on OXPHOS in BMDMs [11,16]. Intact OXPHOS is required for M2 polarization [16,38]. IL-4 induces Akt-dependent increased glycolysis and Akt/mTORC1/ATP-citrate lyase (ACLY) mediated histone acetylation of M2 genes [39]. In addition, IL-4 enhances glucose uptake, glycolysis rate, and glycolytic enzyme expression through STAT6 and PI3K/Akt/mTORC2 mediated IRF4 expression [40]. Glycolysis inhibition by the glucose analogue 2-deoxyglucose (2-DG) suppresses glycolysis, OXPHOS and IL-4 induced M2 polarization [40]. However, 2-DG affects not only glycolysis but also OXPHOS. The inhibition of M2 polarization by 2-DG depends on reduced ATP production followed by impaired STAT6 activation [16]. On the other hand, different glucose concentrations in medium do not affect STAT6 phosphorylation, ATP production, OXPHOS, and M2 polarization [16]. The changes of glycolysis may be compensated for by increased glutamine fueling of Krebs cycle induced by IL-4 [16]. Thus, it is still not clear whether glycolytic reprogramming is required or not for M2 polarization.

## 3. Mitochondrial Fragmentation and Mitophagy

LPS stimulation induces changes of the mitochondrial morphology. The length of mitochondria is much smaller after 2 h of LPS treatment, indicating LPS-induced mitochondrial fragmentation in microglia and macrophages [35,41,42,43,44]. Treatment of Mdivi-1, an inhibitor of mitochondrial fission and fragmentation by targeting dynamin-related protein 1 (Drp1) [45], restores the mitochondrial morphology and attenuates LPS-induced responses, including increased membrane potential, succinate accumulation, the shift of OXPHOS to glycolysis, and the production of ROS and cytokines [35,41,44]. In addition to Mdivi-1 mediated blockage of LPS-stimulated ROS production, ROS seems to participate in LPS-induced mitochondrial fragmentation as the treatment of the ROS scavenger N-acetyl cysteine (NAC) restores LPS-induced mitochondrial fragmentation [42]. The capability of macrophages for clearing up apoptotic cells relies on apoptotic cell-induced mitochondrial fragmentation via Drp1 and is diminished by Mdivi-1 [46]. It should be noted that Mdivi-1 is also reported to have off-target effect including suppressing ROS production by Complex I in non-myeloid cells [47].

Cells utilize mitophagy to clear dysfunctional mitochondria. The smaller size of mitochondria may not trigger damaged mitochondria-induced mitophagy [48]. Nevertheless, mitophagy plays essential roles in IFNγ/LPS-induced macrophage polarization. The mitophagy/autophagy inhibitor 3-methyladenine (3-MA) impairs the expression of glycolytic enzymes, increases mitochondrial mass and reduces pro-inflammatory cytokines induced by IFNγ/LPS [43]. In LPS-primed macrophages, ETC inhibitors (rotenone/antimycin A) cause mitochondria damages followed by mitophagy, resulting in more ROS production [49,50]. In unstimulated macrophages, the treatment of 3-MA induces accumulation of damaged mitochondria and activation of NLRP3 inflammasome as well as increases ROS generation and IL-1β secretion [49], indicating that mitophagy may clear ROS-producing damaged mitochondria to prevent excessive ROS production and inflammation.

## 4. Krebs Cycle

The Krebs cycle (TCA cycle) is an essential process in transforming fuel molecules to energy. It consists of multiple steps converting intermediates: citrate, iso-citrate, α-ketoglutarate (αKG), succinate, fumarate, malate, and oxaloacetate. It generates NADH and FADH_2_ to provide electron for OXPHOS.

During IFNγ/LPS-induced macrophage polarization, two breakpoints of Krebs cycle are induced by IFNγ/LPS [51]. One breakpoint of Krebs cycle is downregulated isocitrate dehydrogenase (IDH), resulting an accumulation of isocitrate and a reduction of αKG [51]. Accumulated citrate is exported from mitochondria into cytosol by the mitochondrial citrate carrier (CIC), which is also up-regulated by LPS stimulation [52]. Then, cytosolic citrate can be converted back to oxaloacetate and acetyl-CoA by ACLY and these acetyl-CoA are utilized in histone acetylation to facilitate inflammatory cytokine expression [13,53]. Meanwhile, IFNγ/LPS increases the conversion of citrate to itaconate [51,54] by up-regulating the enzyme expression (encoded by *Irg1* gene) [51,55,56]. Itaconate is an anti-inflamatory metabolite that inhibits succinate dehydrogenase (SDH)-mediated succinate oxidation [56,57] and activates anti-inflammatory transcription factors Nrf2 and ATF3 [58,59]. These findings indicate a negative feedback regulation after the activation of myeloid cells. The induction of itaconate followed by SDH inhibition also modulate the tolerance of myeloid cells and β-glucan can counteract the tolerance by inhibiting the expression of *Irg1* [60].

Another breakpoint of Krebs cycle occurs at the succinate to fumarate transition [51], leading to the succinate accumulation after LPS stimulation [12,51]. Succinate increases HIF-1α stability and HIF-1α-induced cytokine expression [12]. This accumulation of succinate may be from glutamine metabolism-dependent anerplerosis [61]. Considering that glutamine contributes one third of carbons for accumulated succinate during M1 polarization [51] and a minimum of glutamine (0.03 mM) is required for LPS-induced macrophage activation [62], the fast conversion of glutamine-αKG-succinate may be an essential step to maintain pro-inflammatory responses in macrophages. This point is supported by the evidence that LPS induces higher αKG dehydrogenase (α-KGDH) activity and the αKG/succinate ratio negatively regulates LPS-induced macrophage activation [63]. Moreover, in LPS-induced tolerant macrophages, inhibition of glutamine metabolism to αKG enhances the inflammatory effect of a second LPS stimulation, as well as abolishes LPS tolerance-induced protection from septic shock in mice [63], indicating an anti-inflammatory role of glutamine/αKG in macrophage tolerance.

## 5. Electron Transport Chain

ETC and ATP synthase (also called Complex V) forms the OXPHOS and produces the majority of ATP during aerobic respiration. It consists of four protein complexes (Complex I to IV) and two electron carriers (ubiquinone and cytochrome c). Complex I oxidizes NADH, transfers electrons to ubiquinone (Q) and transport protons from the matrix to the intermembrane space, which results in a proton gradient. Complex II transfers electrons of FADH_2_ originated from succinate to Q without proton transport. Complex III transfers electrons from Q to cytochrome c and transports protons contributing the proton gradient. Complex IV transfers electrons from cytochrome c to the oxygen molecule (O_2_) generating H_2_O and transports protons forming the proton gradient. At last, the proton gradient drives ATP production by ATP synthase.

### 5.1. Mitochondrial Complex I

One of major ROS production sites is through the electron leak at Complex I (CI) during the forward electron transport (FET) and the reverse electron transport (RET) [64]. A highly reduced pool of Q and a large membrane potential (proton gradient) trigger the reverse electron transport (RET) from over-reduced Q back to CI [64,65]. During RET, Q is over-reduced by electrons from Complex II (CII) and other sources such as GPS shunt. The CI inhibitor rotenone blocks the electron transport between CI and Q. During FET, rotenone suppresses the electron transport to Q causing electron leak and increased ROS production in some cell types such as skin fibroblasts [66], while during RET, rotenone prevents the electron transport back from Q leading to reduced ROS production in other cell types such as polarized macrophages [67,68].

In resting myeloid cells, early studies show decreased ROS by rotenone, but a lot more recent evidence indicate the enhanced ROS and pro-inflammatory cytokine production by rotenone (Table 2), suggesting that increased ROS may be resulted from impaired FET in the resting cells. BMDMs have a reduced amount of CI and the decreased association of CI with other mitochondrial respiration complexes in response to bacteria [69], indicating that macrophage activation by infection is associated with lower FET and OXPHOS. In LPS-stimulated myeloid cells, especially after the prolonged treatment (8–24 h), the ROS production and pro-inflammatory cytokine production are reduced by rotenone (Table 2), which may be due to decreased CI mediated RET. Deficiency of the GPS enzyme GDP2 reduces rotenone-induced ROS decrease [13], suggesting that GPS-mediated Q reduction also contributes to RET after LPS stimulation.

Besides pharmaceutical inhibition, genetic models of CI are also utilized. CI is a supercomplex of 45 subunits which form three modules: N module (oxidizing NADH and electron input), Q module (electron output to ubiquinone) and P module (proton transport) [86]. Deficiency of CI accounts for about 30% of mitochondria disorders including Leigh syndrome and lactic acidosis [86]. Ndufs4 is a subunit of N module and its deletion in mice leads to a crippled CI without N module and decreased CI activity [87,88], indicating defective electron input and FET for OXPHOS. Ndufs4 knockout (KO) mice die early at about week 7, and Ndufs4 KO neonates show fur loss during week 2–4 [88,89,90,91]. Our group demonstrates that fur loss of global Ndufs4 KO mice is associated with a systematic inflammation including elevated pro-inflammatory cytokines in serum and expression of pro-inflammatory markers in cells from bone marrow, spleen, liver and skin together with the accumulation of monocytes/macrophages [91]. Ndufs4 KO BMDMs produce more lactate and ROS than wild-type (WT) BMDMs [91], indicating that CI deficiency leads to inflammatory responses and metabolic shift to glycolysis. CI deficiency induces 50% higher serum triglyceride and nonesterified fatty acid in Ndufs4 KO suckling neonates and this effect is due to defective fatty acid metabolism in liver [91]. Saturated fatty acids activate TLR2/4-mediated inflammation [92]. Indeed, Ndufs4+TLR2/4 triple KO rescues systemic inflammation in Ndufs4 KO mice [91], suggesting CI deficiency causes inflammation through both increased ROS in macrophages and defective fatty acid metabolism in the liver.

Another mouse model for studying CI is macrophage KO of ECSIT (evolutionarily conserved signaling intermediate in Toll pathways), a CI assembly protein [93,94]. ECSIT deletion leads to less CI, elevated glycolysis, impaired OXPHOS, reduced mitochondrial potential and increased ROS in unstimulated macrophages [94]. After TLR1/2/4 activation, TRAF6 mediates ECSIT ubiquitination and enrichment leading to ROS production, and ECSIT deletion in LPS-stimulated BMDMs results in reduced ROS and anti-bacteria response [74]. ECSIT forms a complex with TAK1 and TRAF6 in human monocyte THP-1 cells to enhance LPS-induced TLR4 signals [95]. Overall, the suppression of CI induces inflammation in unstimulated myeloid cells and impairs inflammation in stimulated myeloid cells.

### 5.2. Complex II and III

The activity of CII in myeloid cells is enhanced by inflammatory signals. LPS stimulation induces the oxidation of accumulated succinate by the increased activity of SDH (another name of CII) and increased mitochondrial membrane potential followed by mitochondrial ROS production and the cytokine expression [24]. Similarly, bacteria promote the activation of CII and the inflammatory responses in BMDMs [69].

CIII is another site of ROS generation in mitochondria. Myxothiazol (Myx) is a reversible CIII inhibitor that suppresses the electron transfer to CIII at the ubiquinol oxidation center Q_o_ site, while antimycin A inhibits CIII by blocking Q turn over at the ubiquinone-reduction center Q_i_ site [96]. In unstimulated BMDMs and bone marrow neutrophils, antimycin A or Myx decreases membrane potential and increases ROS production [72,96,97]. In BMDMs stimulated by IFNγ/LPS for 1 h, Myx blocks the IFNγ/LPS-induced ROS production [14]. Interestingly, removal of the revisable inhibitor Myx after the first hour of LPS treatment is still able to diminish production of pro-inflammatory cytokines after LPS treatment for 18 h [14]. Moreover, Myx and antimycin A reduce NFκB nuclear accumulation after LPS treatment for 1 h and decrease the pro-inflammatory cytokine production after LPS treatment for 5 h in bone marrow neutrophils [97]. All these findings indicate a similar role of CIII as CI that suppressing CIII induces inflammatory responses in unstimulated myeloid cells and blocks inflammatory responses in LPS-stimulated myeloid cells, which may be associated with diminished FET and RET, respectively.

## 6. Fatty Acid Oxidation and Synthesis

IFNγ/LPS-induced M1 macrophages have increased fatty acid synthesis (FAS) [98,99,100]. FAS in M-CSF derived hMDMs is dramatically enhanced by the up-regulation of sterol regulatory element-binding transcription factor 1c (SREBP1c) target genes involved in FAS, such as fatty acid synthase [100]. Knocking down or inhibition of fatty acid synthase in LPS-stimulated BMDMs decreases the expression of pro-inflammatory cytokines [101]. Fatty acid synthase regulates cholesterol synthesis via the FAS intermediate metabolite acetoacetyl-CoA, which is also a metabolite in the cholesterol synthesis [101]. The cholesterol level regulated by FAS maintains the cholesterol-rich lipid rafts where TLR4 translocates in inflammation signaling [101].

Fatty acid oxidation (FAO) of M1 macrophages is decreased via suppressing the expression of CPT-1 [38,98], which transports fatty acid into mitochondria. Fatty acid is converted to acetyl-CoA, NADH and FADH_2_ by β-oxidation to fuel OXPHOS. Interestingly, glucose deprivation leads to reduced glycolysis and enhanced OXPHOS after LPS treatment as compared to normal glucose supplement [19]. The enhanced OXPHOS in the absence of glucose is due to β-oxidation of fatty acid, as inhibition of fatty acid transport into mitochondria by 250 μM of the CPT-1 inhibitor etomoxir attenuates glucose deprivation-induced OXPHOS [19]. However, high doses of etomoxir (>100 μM) have multiple off-target effects including inhibiting the adenine nucleotide translocase (ANT) and mitochondrial Complex I [102].

IL-4-induced M2 macrophages have increased FAO [38,103]. M2 macrophages and myeloid-derived suppressor cells (MDSCs) have increased OXPHOS which is suppressed by 100–200 μM of etomoxir [16,103,104]. However, another study shows that FAO may be dispensable for hMDMs, as IL-4 does not induce CPT-1 expression and 10 μM etomoxir is sufficient to inhibit FAO, but not IL-4-induced polarization [105]. Conditional KO of CPT-2 in macrophages blocks FAO, but has no effect on M2 macrophage polarization [106]. Considering the possible off-target effects of etomoxir, FAO may participate but is dispensable in maintaining OXPHOS in IL-4-induced macrophage polarization

## 7. Amino Acid Metabolism

Glutamine deprivation does not affect IFNγ/LPS-induced M1 polarization [51], but it dramatically suppresses IL-4-induced M2 polarization [51,63]. Glutamine provides one third of all carbons for metabolites in the Krebs cycle and supports the expression of chemokines in IL-4-induced M2 macrophages [16,51,63]. However, glutamine deprivation is also reported to elevate LPS-induced macrophage activation [63]. Glutamine can replenish Krebs cycle metabolite αKG, which is reduced during M1 polarization [51], via glutaminolysis. Inhibition of glutaminolysis enhances, while supplement of αKG impairs, LPS-induced macrophage activation [63], suggesting glutamine and αKG may have anti-inflammatory effect.

However, *M. tuberculosis* infection in human leads to an up-regulated expression of the glutaminolysis enzyme GLUD2 and increased αKG in macrophages [107]. Glutamine deprivation and inhibition of glutamine uptake or glutaminolysis decrease the *M. tuberculosis*-induced pro-inflammatory cytokine production in PBMCs [107].

Besides glutamine, aspartate is also involved in mitochondrial metabolic reprogramming during the activation of myeloid cells. The aspartate-argininosuccinate shunt (AASS) replenishes the malate pool of Krebs cycle, and inhibition of this shunt blocks the shift from OXPHOS to glycolysis and IFNγ/LPS-induced M1 polarization [51].

## 8. Concluding Remarks and Future Directions

**Figure 1:**
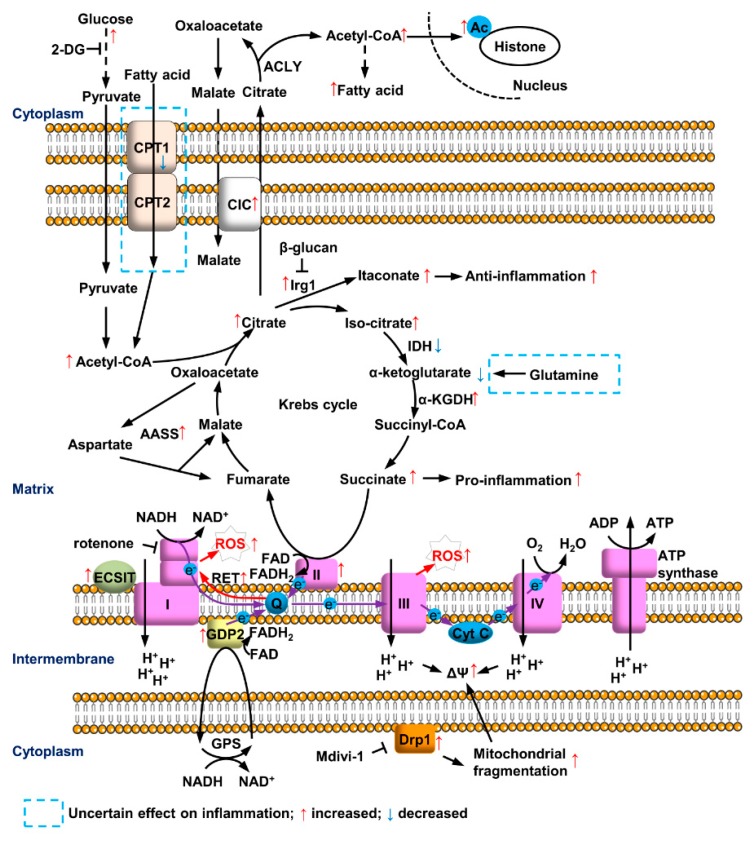
Mitochondrial metabolic reprogramming in myeloid cells after LPS treatment. LPS induces glycolysis and accumulation of citrate and succinate in Krebs cycle. Citrate triggers pro-inflammation by metabolizing to Acetyl-CoA and anti-inflammatory feedback regulation via converting to itaconate. Accelerated succinate oxidation at CII and GDP2-mediated GPS increase the reduction of ubiquinone (Q), leading to ROS production at CI and CIII of ETC. Fatty acid synthesis, AASS and mitochondrial fragmentation are involved, while the roles of fatty acid oxidation and glutamine metabolism are still not clear.

**Figure 2:**
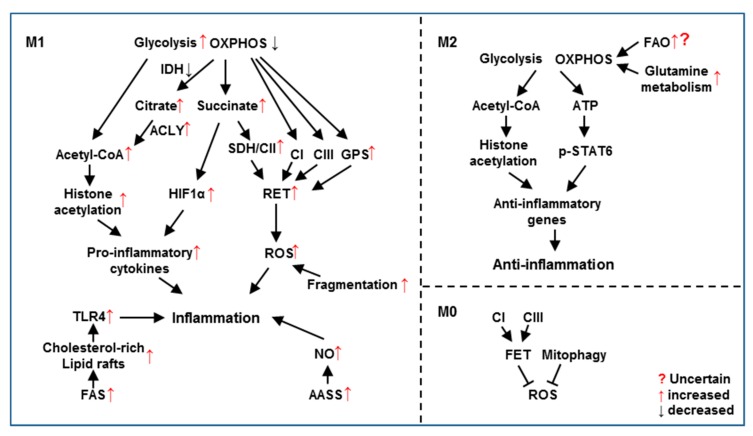
Mitochondrial metabolic reprogramming regulates functions of M1 and M2 macrophages. In M1 macrophages, increased glycolysis and decreased OXPHOS modulate the pro-inflammatory response through elevating histone acetylation, HIF1α and RET. Increased FAS, AASS and mitochondrial fragmentation also contribute to M1 polarization. In M2 macrophages, intact glycolysis and OXPHOS are required for anti-inflammatory response by inducing histone acetylation and STAT6 phosphorylation. Glutamine metabolism is essential to replenish Krebs cycle while FAO’s role is uncertain for M2 polarization. In M0/unstimulated macrophages, inhibition of CI or CIII blocks FET followed by ROS production. Mitophagy removes ROS-producing damaged mitochondria.

This review aims to summarize the current understandings of the critical roles of mitochondrial metabolic reprogramming for immune responses of myeloid cells. To perform their pro- or anti-inflammatory functions, myeloid cells adapt their metabolism by involving changes in almost every metabolic step (Figure 1). These cell-type specific metabolic changes then modulate immune responses of myeloid cells such as M1 and M2 macrophages (Figure 2). Most of the enzymes or metabolites have been extensively studied to exhibit a whole picture of metabolic reprogramming with a clear view of most parts and some fuzzy spots. These fuzzy spots need further investigation. Despite the exciting advancement of immunometabolism, there are still plenty of challenges waiting for immunologists. The dose, ligand and cell type-dependent effects request careful examinations to determine a more physiological relevant stimulation condition. The metabolic alterations in vitro may be quite different from those in vivo, but only limited in vivo models are available. Making the situation even more challenging, many enzyme inhibitors have off-target effects, for example, 2-DG, etomoxir and Mdivi-1 as discussed above. Genetic animal models should provide more specific effect. As genetic deficiency of metabolism affects development especially of the nerves system, it is better to use the conditional knockout by Cre expressed specifically in myeloid cells. Inducible gene expression or knockout is another option to establish in vivo models. Technology advancement will also benefit immunometabolism. For example, cryo-EM can reveal protein and organelle structures and single cell sequencing can demonstrate the diversity of immune responses. A better understanding of the complicated metabolic reprogramming process in immune cells will facilitate the discovery of new strategies to treat diseases related to immunity.

## Figures and Tables

**Table 1:** The effect of LPS on glycolysis and OXPHOS.

LPS (ng/mL)	Treatment Period (hour)	Cell Type	Glycolysis	OXPHOS	Pro- Inflammatory Cytokines	Year	Reference
**Early response**							
100	0–2	BMDMs	↑	↓	↑	2012	[11]
100	0–4	RAW 264.7	↑	↓			
100	4	BMDMs	↑	↑	↑	2013	[12]
100	1	BMDMs		↑		2019	[13]
100	0.5, 1	BMDMs		↑		2019	[14]
100	0–2	BMDMs	↑	n.c.		2016	[15]
100	0–2	hMDMs	↑	n.c.			
100	0–2	BMDMs	↑	n.c.		2018	[16]
100	0–4	Peritoneal mΦ	↑			2010	[17]
10	4	Monocytes	↑	↑		2016	[18]
100	0–1	Monocytes	↑	↑		2017	[19]
100	0–6	BMDCs		↑		2012	[20]
100	0–2	BMDCs	↑	n.c.		2014	[21]
**Sustained response**							
100	8, 16, 24	BMDMs	↑	↓	↑	2013	[12]
100	24	BMDMs	↑	↓	↑	2015	[22]
100	24	BMDMs	↑	↓		2016	[23]
100	24 or 48	BMDMs	↑	↓	↑	2016	[24]
100	24	BMDMs	↑	↓		2016	[15]
10	12	BMDMs	n.c.	↑		2016	[25]
100	12	BMDMs	↑	↓			
5000	12	BMDMs	↑	↓			
100	24	BMDMs	↑	↓	↑	2017	[26]
100	4–24	BMDMs		↓	↑	2019	[13]
1000	16	mBMDMs	↑	↓	↑	2019	[27]
1000	16	hMDMs	↓	n.c.	↑		
100	4–12	Peritoneal mΦ	↑		↑	2010	[17]
10	24	Monocytes	↑		↑	2014	[28]
1–100	24	Monocytes	↑	↓	↑	2016	[18]
0.1	24	Monocytes	↑	↑	↑		
10	4 or 24	PBMCs	↑	↓	↑	2016	[29]
10	18	RAW 264.7	↑	↓		2017	[30]
100	24	Neutrophils	↑	↓		2019	[31]
100	24	BMDCs	↑	↓		2010	[32]
100	6-24	BMDCs	↑	↓	↑	2012	[20]
100	24	BMDCs	↑	↓		2014	[33]
1000	24	BMDCs	↑	↓		2019	[34]
50	6 or 24	Microglia	↑	↑		2019	[35]
100	6	Microglia	↑	↑			
100	24	Microglia	↑	↓	↑		
**Tolerant response**							
First LPS (ng/mL) for period (hour)	Second LPS (ng/mL) for period (hour)						
100 for 24	10 for 4	BMDMs		↓ *	↓ *	2019	[13]
100 for 24	10 for 24	hMDMs			↓ *	2014	[36]
10 for 24	10 for 24	Monocytes	↓ *	↓ *		2016	[29]

* compared with non-tolerant cells; BMDMs, bone marrow-derived macrophages; hMDMs, human monocyte-derived macrophages; BMDCs, bone marrow-derived dendritic cells; PBMCs, peripheral blood mononuclear cells; mΦ, macrophages; n.c. no change; ↑ increased; ↓ decreased.

**Table 2:** The effect of Complex I inhibitor rotenone on unstimulated or LPS-stimulated myeloid cells.

Rotenone (μM)	Rotenone Treatment	LPS (μg/mL) for Period	Cell Type	ROS Method	mROS	Pro- Inflammatory Cytokines	Year	Reference
**Single Agent**								
2	0–30 min		Alveolar mΦ	LDCL	↓		1994	[70]
0.1–10	0–30 min		ML1-M	LDCL	↓		1998	[68]
0.2–5	30 min		HL-60	PHPA	↑		2003	[71]
10	90 min		BMDMs	DCFH_2_-DA	↑		2008	[72]
10	6 h		THP-1	MitoSOX	↑	↑	2011	[49]
5	30 min		J774A.1	MitoSOX	↑		2011	[73]
0.5	16 h		RAW 264.7	MitoSOX	↑		2011	[74]
0.01	30 min		Peritoneal mΦ	WST-1	↑		2012	[75]
0.01	30 min		Microglia	WST-1	↑		2012	
0.001–0.1	6 h		BV2 microglia	DCFH-DA	↑	↑	2013	[76]
1	6 h		BV2 microglia	DCFH-DA	↑	↑	2013	[77]
0.01	18 h		Microglia	DCFH-DA	↑		2014	[78]
0.5	25 h		BMDMs	CellROX	n.c.		2015	[79]
5	2 h		BMDMs	MitoSOX	↑	n.c.	2015	[80]
1	1.5 h		BMDMs	MitoSOX	↑		2016	[69]
10	0–30 min		ML1-M	LDCL	↓		2016	[81]
unknown			mΦ from THP-1	DCFH-DA	↑		2017	[82]
2	30 min		hMDMs	MitoSOX	↑		2019	[27]
1.5	0–100 s		BMDMs	MitoSOX	↑		2019	[13]
**Co-treatment**								
1	18 h	1 for 18 h	J774.1	DCFH-DA	↓	↓	2000	[83]
5	10 min + 30 min	0.1 for 30 min	RAW 264.7	DCFH-DA/LDCL	↓		2004	[84]
0.5	1 h + 24 h	1 for 24 h	BMDMs	CellROX	↓	↓	2015	[79]
0.5	3 h + 24 h	1 for 24 h	BMDMs	CellROX	↓	↓	2016	[24]
0.1	1 h + 8 h	1 for 8 h	RAW 264.7	DCFH_2_-DA	↓	↓	2018	[85]
1.5	0–100 s	0.1 for 12 h	BMDMs	MitoSOX	↓		2019	[13]

mΦ, macrophages; mROS, mitochondrial ROS; ML1-M, ML-1-derived monocytes/macrophages; LDCL, Lucigenin-derived chemiluminescence; s, second; min, minute, h, hour; n.c. no change; ↑ increased; ↓ decreased.

## Abbreviations

LPS, lipopolysaccharide; IFNγ, Interferon gamma; TLR, Toll-like receptor; ETC, electron transport chain; FET, forward electron transport; RET, reverse electron transport; OXPHOS, oxidative phosphorylation; ROS, reactive oxygen species; CI, mitochondrial Complex I; CII, mitochondrial Complex II; CIII, mitochondrial Complex III; mΦ, macrophages; DCs, dendritic cells; BMDMs, bone marrow-derived macrophages; hMDMs, human monocyte-derived macrophages; PBMCs, peripheral blood mononuclear cells; BMDCs, bone marrow-derived dendritic cells; n.c. no change; HIF1α, Hypoxia-inducible factor-1α; mTOR, mammalian target of rapamycin; AMPK, AMP-activated protein kinase; ATP, adenosine triphosphate; NADH, nicotinamide adenine dinucleotide; FADH_2_, flavin adenine dinucleotide reduced; TCA cycle, tricarboxylic acid cycle; ECAR, extracellular acidification rate; OCR, oxygen consumption rate; SRC, spare respiratory capacity; GPS, Glycerol 3-phosphate shuttle; GDP2, mitochondrial glycerol 3-phosphate dehydrogenase; iNOS, inducible nitric oxide synthase; NO, nitric oxide; FAS, fatty acid synthesis; FAO, fatty acid oxidation; CPT-1, Carnitine Palmitoyltransferase 1; MDSCs, myeloid-derived suppressor cells; TBK1, TANK-binding kinase 1; IKKε, IκB kinase ε; KO, knockout; P3C, Pam_3_CysSK_4_; poly(I:C), polyinosinic:polycytidylic acid; R848, Resiquimod; CpG, CpG oligodeoxynucleotides; NAC, N-acetyl cysteine; NLRP3, NACHT, LRR and PYD domains-containing protein 3; 3-MA, 3-methyladenine; αKG, α-ketoglutarate; ACLY, ATP-citrate lyase; SDH, succinate dehydrogenase; NRF2, Nuclear factor erythroid 2-related factor 2; ATF3, Activating transcription factor 3; Q, ubiquinone; WT, wild-type; ECSIT, evolutionarily conserved signaling intermediate in Toll pathways; TRAF6, tumour necrosis factor receptor-associated factor 6; Myx, Myxothiazol; TAK1, TGF-beta-activated kinase 1; SREBP1c, sterol regulatory element-binding transcription factor 1c; GLUD2, Glutamate dehydrogenase 2; 2-DG, 2-deoxyglucose; IRF4, Interferon Regulatory Factor 4; IDH, isocitrate dehydrogenase; Drp1, dynamin-related protein 1; ANT, adenine nucleotide translocase; α-KGDH, α-ketoglutarate dehydrogenase; AASS, aspartate-argininosuccinate shunt; ΔΨ, membrane potential; ML1-M, ML-1-derived monocytes/macrophages; LDCL, Lucigenin-derived chemiluminescence; mROS, mitochondrial-derived reactive oxygen species; CIC, the citrate carrier; Cyt C, cytochrome c; Cryo-EM, cryogenic electron microscopy.
